# Effects of crude oligosaccharide extract from agricultural by-products on the performance and gut development of broilers

**DOI:** 10.5713/ab.22.0338

**Published:** 2023-01-11

**Authors:** Janjira Sittiya, Suphavadee Chimtong, Phumipat Sriwarcharameta

**Affiliations:** 1 Faculty of Animal Science and Agricultural Technology, Silpakorn University, Phetchaburi 76120, Thailand

**Keywords:** Histology, Nutrient Digestibility, Oligosaccharides, Performance, Prebiotics

## Abstract

**Objective:**

This study aimed to determine the effect of crude oligosaccharide extract from coconut milk meal (CMM) and spent tea leaves (ST) on the performance and gut development of broiler chickens.

**Methods:**

A total of 240 one-day-old unsexed broiler chicks (ROSS 308) were raised on litter-floored pens and had *ad libitum* access to water for 42 days. The experiment was conducted on chicks fed with basal diet (CON), commercial mannan-oligosaccharides (MOS), crude oligosaccharide extract from CMM, and crude oligosaccharide extract from ST. The experimental diets were supplemented with 2 and 1 g/kg oligosaccharides during the starter and grower periods, respectively.

**Results:**

The body weight gain (BWG) of birds in the MOS group was higher than that of birds in the other groups (p<0.05) in the starter period. However, during the grower period, ST significantly improved the BWG compared to the MOS (p<0.05). MOS, CMM, and ST showed no influence on the carcass and visceral organ weight and the weight and length of intestine (p>0.05). The digestibility of gross energy was greater (p<0.05) in the CMM group than in the CON group during the grower period. Morphological changes were absent in the dietary treatments (p>0.05).

**Conclusion:**

The improvements in the growth performance were partly driven by nutrient digestibility of such oligosaccharides having prebiotic properties. This result can indicate that supplementing broiler diets with crude oligosaccharides from CMM and ST had no negative effect on the growth performance and gut development of broilers.

## INTRODUCTION

Antibiotics as feed additives have been used in the livestock industry for economic benefits and enhancement of growth performance. However, the use of antibiotics as a growth promoter has been a concern due to the development of microbial resistance [1]. In this scenario, dietary prebiotics are one of the alternatives for growth promoters. Prebiotics are non-digestible compounds that provide beneficial physiological effects on the host by stimulating the activity of gut microbiota [2]. Natural and synthetic oligosaccharides are known types of prebiotics, and they can be used in poultry as an alternative to antibiotic growth promoters.

Oligosaccharides are carbohydrates and composed of short chains of monosaccharides; they improve the performance of poultry, enhance growth of beneficial microbiota in the gut, and stimulate immune ability [3–5]. In addition, several studies have been carried out to evaluate the feeding of oligosaccharides as prebiotics to poultry. Oligosaccharides can improve feed efficiency due to the enhancement of the intestinal morphology [6].

In Thailand, coconut milk is used for food and sweets. Coconut milk meal (CMM) is a by-product of coconut milk, and it contains approximately 43% to 45% of carbohydrates [7,8]. Spent tea leaves (ST) are a by-product from tea beverage shops; they contain 27.63% crude protein, 43.92% neutral detergent fiber, and 26.34% acid detergent fiber [9]. Both by-products contain high amounts of natural fibers. However, a limited number of studies reported the use of these by-products in broiler diets. Therefore, the use of CMM and ST should be maximized to increase their residual value. As these residual fibers mainly consist of cellulose, hemicellulose and lignin they can be pre-treated with several methods for oligosaccharide production [10–12]. Oligosaccharides can be produced by chemical, enzymatic, hydrothermal, or a combination of chemical and enzymatic methods from coconut meal and ST [13,14]. Some researchers showed that commercial mannan-oligosaccharides (MOS) can effectively suppress enteric pathogens, enhance the immune response, and improve the intestinal mucosa in broilers [15,16]. Moreover, Xu et al [17] reported that supplementation of 8 g/kg fructo-oligosaccharides (FOS) improved the performance and increased the levels of cecal lactobacilli and bifidobacteria in broiler chickens. This finding is in accordance with the results of Catalá-Gregori et al [18], who observed an improved growth performance and intestinal physiology in broilers fed with sugar beet short-chain FOS. In addition, the oligosaccharide extract from palm kernel expeller improved immune responses in broiler chickens at a young age [5]. The effects of feeding commercial oligosaccharides with prebiotic properties to broilers have been extensively reviewed by several reports. However, literature reviews on the use of crude oligosaccharide extract from agricultural by-products as prebiotics in broilers are scarce. Therefore, this study aimed to determine the effect of crude oligosaccharide extract from CMM and ST on the performance and gut development of broiler chickens.

## MATERIALS AND METHODS

### Preparation of crude oligosaccharides from agricultural by-products

The agricultural by-products used in this study were CMM and ST. CMM and ST were obtained from the local market and tea shops, respectively. The samples were dried in oven at 60°C until a constant weight was obtained and then ground in a hammer mill (RT-34 model; Rong Tsong Precision Technology Co., Taichung, Taiwan) equipped with a 250 μm screen.

Crude oligosaccharides were extracted from the dried sam ples. Briefly, all dried samples were stirred continuously in distilled water with 1 N HCl for 2 h using a magnetic stirrer with a heating plate (IKAMAG C-MAG HS7; IKA-Werke GmbH&Co.KG, Staufen, Germany) at 100°C. After cooling down, the sample pH was adjusted to 7.0 using Ba(OH)_2_. Then, the samples were further hydrolyzed by 5 g/kg Hemicell enzyme (Behn Meyer Chemical Co., Ltd., Bangkok, Thailand) at 40°C for 24 h. The insoluble materials were removed by centrifugation at 10,000 rpm×5 min. Subsequently, the supernatant was precipitated with 90% acetone and then kept overnight at 4°C in a refrigerator to precipitate crude oligosaccharides [19]. Crude oligosaccharides were obtained after oven-drying the precipitate at 50°C.

### Animals and diets

The experiment was carried out following the guidelines and rules for animal experiments of the Faculty of Animal Sciences and Agricultural Technology, Silpakorn University, Thailand (ID Project 21/2562). A total of 240 one-day-old unsexed broiler chicks (ROSS 308) were obtained from a commercial hatchery. The chicks were weighed individually and randomly divided into four groups of chicks with similar mean body weights, with each group having five replicates of twelve chickens with a pen floor area of 1 m^2^ per bird. The chicks were housed in litter-floored pens with rice hulls under continuous lighting and had *ad libitum* access to water for 42 days.

Commercial starter (0 to 21 days) and grower (22 to 42 days) diets were used as basal diets in this experiment. The nutrient contents of the experimental diets were met or exceeded by Aviagen [20] and the same ratio of metabolisable energy to crude protein applied in all diets (Table 1). The five dietary treatments were as follows: i) basal diet only (CON), ii) basal diet supplemented with MOS (NANOMOS; Specialty Biotech Co., Ltd., Chonburi, Thailand), iii) basal diet supplemented with CMM, and iv) basal diet supplemented with ST. The experimental diets were supplemented with 2 and 1 g/kg crude oligosaccharides during the starter and grower periods, respectively. All diets were provided to the broilers in mashed form.

### Growth performance and digestive organ development

The feed intake (FI), body weight gain (BWG), and mortality of the chicks in each replicate cage were recorded after 21 and 42 days. Whenever a bird was found dead, the feed and the dead bird were weighed immediately. The FI and BWG of the dead bird can be calculated and deducted from the final FI and BWG of its cage, respectively. The feed conversion ratio (FCR) was also calculated.

At the end of the feeding experiment, five birds of a cer tain average body weight (ranging between±100 g of the average body weight) from each group were used to determine digestive organ development and carcass traits. The digestive organs were carefully removed. The lengths of duodenum, jejunum, ileum, and ceca were measured individually. Subsequently, the weights of the proventriculus, gizzard, duodenum, jejunum, ileum, and ceca were recorded after the digesta content had been removed. The weight of empty organs was expressed relative to 100 g body weight.

### Tissue sampling for intestinal morphological observation

At 42 days of age, another five birds per group were used for intestinal morphological observations. Immediately following decapitation, the midpoint of each intestinal segment (duodenum, jejunum, and ileum) was removed and fixed in 10% neutral-buffered formalin. After dehydration through varying concentrations of alcohol, each intestinal segment was embedded in paraffin wax. A 4 μm-thick transverse section was cut and stained with hematoxylin–eosin. Subsequently, measurements were conducted using the Toup View 3.7 software (AmScope, Irwin, CA, USA). The villus height, villus area, and crypt depth were measured using a light microscope as described by Iji et al [21]. Eight calculations from eight sections showing the villus height, villus area, crypt depth, and villus height/crypt depth ratio were averaged for each bird.

### Nutrient digestibility

A digestibility study was conducted using 3% chromic oxide (Cr_2_O_3_) as an indicator and observed during the last week of two periods (during weeks 3 and 6 of the experiment). On day 14, four birds from each group were moved to individual cage for digestibility study. The chickens were fed experimental diets mixed with Cr_2_O_3_. Feces from each replication were collected over a 24 h period on day 21. All feed and fecal samples were stored immediately at −20°C until analysis. The procedure was repeated on day 35 and feces were collected on day 42. All samples were finely ground and analyzed for gross energy using a bomb calorimeter (Parr 6200 calorimeter; Parr Instrument Company, Moline, IL, USA). The concentration of Cr_2_O_3_ in the feed and feces was determined by the method described by Fenton and Fenton [22]. The nutrient digestibility was calculated according to the following equation:


$$\text{Digestibility }(\%)=100-100\times [({\text{Cr}}_{2}{\text{O}}_{3}\;\text{Diet}\times \text{Nutrient Feces})/({\text{Cr}}_{2}{\text{O}}_{3}\;\text{Feces}\times \text{Nutrient Diet})]$$

### Statistical analysis

Data from the experimental groups were statistically analyzed using one-way analysis of variance in SPSS statistical software (version 19.0; IBM Corp. Armonk, NY, USA). Significant differences among the treatments were determined using Tukey’s test. Statistical significance was accepted at p< 0.05.

## RESULTS

### Growth performance and digestive organ development

Table 2 shows the effects of crude oligosaccharide extract from agricultural by-products on the growth performance of broilers. During the starter period, the BWG of birds in the CMM, ST, and CON groups showed no difference (p> 0.05), whereas the BWG of birds in the MOS group was higher than those of the other groups (p<0.05). However, no significant difference was observed in the FI, FCR, and mortality. During the grower period, the ST significantly improved the BWG compared with the MOS (p<0.05). The FI, FCR, and mortality of birds were not affected by dietary treatments (p>0.05). During the entire experimental period, the FI, BWG, FCR, and mortality did not differ (p>0.05) among the groups.

Tables 3 and 4 show the carcass and visceral organ weight in the 42-day-old broiler chickens fed with diets supplemented with crude oligosaccharide extract from agricultural by-products. No difference was observed in the carcass traits and visceral organ weight of the treatment groups.

### Intestinal morphology

As shown in Table 5, no significant differences were observed in the villus height, villus area, crypt depth, and villus height/crypt depth ratio of duodenum, jejunum, and ileum in any treatment. However, the villus height/crypt depth ratio of duodenum tended (p = 0.051) to be higher in the ST and MOS groups compared with the CON group.

### Nutrient digestibility

No significant differences in the digestibility of dry matter and gross energy were found in any treatments during the starter period (Table 6). However, the digestibility of gross energy was greater (p<0.05) in the CMM group than in the CON group during the grower period.

## DISCUSSION

Several commercial oligosaccharide supplementations have been reported to improve growth performance, gut development, and immune responses [23–25]. To our knowledge, less is known about the effect of crude oligosaccharide extracts from agricultural by-products on chickens. This research is the first to determine the effect of CMM and ST on the performance and gut development of chickens. For comparison, we used MOS as the positive control to investigate the efficiency of crude oligosaccharide extract from agricultural by-products. The current study revealed that the BWG improved in birds fed with the MOS diet compared with those fed with other diets in the starter period. Meanwhile, in the grower period, a better BWG was observed in birds fed with ST compared with the other groups. Hooge [26], based on a meta-analysis of 44 research experiments with broilers, reported that chickens fed with MOS showed enhanced growth performance and feed efficiency compared with those fed with Antibiotic growth promoter-free diets. Moreover, Rezaei et al [5] reported that supplementing oligosaccharide extract from palm kernel expeller (OligoPKE) had no effect on average daily gain and average daily FI throughout the experimental period; however, the birds fed with OligoPKE had better gain-to-feed ratio during the finisher and overall periods.

During the hydrolysis of non-starch polysaccharides, small oligosaccharides, which have prebiotic properties, are generated [27]. These oligosaccharides improve the growth performance of broiler chicks by increasing nutrient absorption due to modulation of gut microflora and improved gut integrity [28]. This finding may explain the improved BWG of broilers fed with crude oligosaccharides as prebiotics in the current study. However, further studies are needed to improve our knowledge of the influence of crude oligosaccharides on higher number of replicates and birds.

In the current study, the nutrient digestibility of dry matter tended to be higher in birds given MOS than the other groups during the starter period. In addition, the results of this study also confirmed the enhanced digestibility of gross energy in the CMM dietary treatment groups during the grower period. These results are also supported by those of other studies, which reported that the improved BWG of birds fed with MOS diets may be explained by binding the sites attacked by pathogenic bacteria on the intestinal mucosa and decreasing the intestinal injury; consequently, the cellular turn over results in the greater utilization of such diets [29]. Additional evidence of the benefits of prebiotics can be found in several studies which indicated that after ingestion of prebiotics, the nutrient digestibility was enhanced in broilers [23]. Tuohy et al [30] also reported that the improved nutrient digestibility in birds fed with oligosaccharides was due to enhanced gut health. This finding may explain the increase in the nutrient digestibility observed as oligosaccharide supplementation reduced the number of pathogenic bacteria and increased the number of beneficial bacteria in the small intestine [4,17], resulting in the improved digestion of nutrients. Therefore, further studies are needed to confirm whether the effect of CMM and ST on nutrient digestibility is due to the gut microflora or other factors.

The intestine has a close relationship between digestion and absorption, requiring an intestinal histology approach that may be able to access the intestinal function [31]. This is in agreement with the finding of Caspary [32], who reported that greater villus height contributes to an increased surface area for greater absorption of available nutrients. Xu et al [17] also reported that short villi and deep crypts can lead to a decrease in the growth performance and poor nutrient absorption. The morphological alterations of the small intestine are indicators of an activated villus function [33]. These morphological changes were not found in dietary treatments except that the villus height/crypt depth ratio of duodenum tended to be higher in the ST and MOS groups compared with the CON group. This may be due to oligosaccharides being hydrolyzed to short chain fatty acids, which are a source of energy for enteric mucosa and a stimulant of villi growth [34]. In another study of broilers, the diet containing FOS increased the ileal villus height, jejunal and ileal microvillus height, and villus height-to-crypt depth ratios in the jejunum and ileum [17]. In addition, the dietary MOS increased the villus width and height in the jejunum and ileum of quail breeders [35]. These discrepancies may be caused in part by the different structures, contents, or doses of oligosaccharides used in the experiment. The functional properties of oligosaccharides from different sources vary due to their variable monomers, degree of polymerization, and osidic bonds [36]. Therefore, further studies are needed to confirm their structural characterization.

In the current study, MOS, CMM, and ST showed no in fluence on the carcass and visceral organ weight and the weight and length of intestine. A non-stressful condition may be the reason for the lack of an effect of these substances on the carcass and gut parameters. Most beneficial additives exhibit their maximum effect under disease and stress conditions [26]. Therefore, the lack of a significant effect of all the oligosaccharides used in this study on carcass and gut parameters can be due to the ideal environmental condition during the entire experimental period.

## CONCLUSION

The improvements in the growth performance were partly driven by higher nutrient digestibility due to oligosaccharides having prebiotic properties. In overall, current result indicates that supplementing broiler diets with crude oligosaccharides from CMM and ST has no negative effect on the growth performance and gut development.

## Figures and Tables

**Table 1:** Feed compositions and calculated nutrient value of experimental diets

Item	Starter period (0 to 21 days of age)	Grower period (22 to 42 days of age)
Ingredient (%)		
Corn	41.32	50.97
Soybean meal	40.37	32.60
Soybean oil	7.73	6.12
Rice bran	5.00	5.00
Monocalcium phosphate	2.00	1.79
Limestone	1.30	1.16
Premix^1)^	0.65	0.60
Salt	0.40	0.40
D, L-methionine	0.17	0.16
Choline chloride	0.80	0.80
L-lysine	0.04	0.11
L-threonine	0.02	0.04
Calculated contents		
Crude protein (%)	23.00	20.00
Metabolizable energy (kcal/kg)	3,200	3,200
Crude fiber (%)	3.10	2.90
Crude fat (%)	10.30	9.00
Ash (%)	5.80	5.20
Calcium (%)	1.00	0.90
Available phosphorus (%)	0.50	0.50
Available lysine (%)	1.10	1.00
Available methionine (%)	0.40	0.40

1) Premix included the following (per kg of diet): retinol, 2.48 mg; cholecalciferol, 0.07 mg; tocopherol, 20.11 mg; menadione, 1.1 mg; thiamine, 1.4 mg; riboflavin, 5.5 mg; pyridoxine, 1.1 mg; cyanocobalamin, 12 μg; niacin, 41.3 mg; pantothenic acid, 11 mg; biotin, 41 μg; folic acid, 1.4 mg; manganese, 125 mg; iron, 282 mg; copper, 27.5 mg; zinc, 275 mg; iodine, 844 μg; selenium, 250 μg.

**Table 2:** Growth performance in broiler chickens fed diets supplemented with crude oligosaccharides extract from agricultural by-products during 0–42 days old (n = 5)

Items	Dietary group^1)^				SEM	p-value

	CON	MOS	CMM	ST		
Starter period (1 to 21 d)						
Feed intake (g)	1,318.80	1,250.00	1,246.60	1,266.40	33.05	0.879
Body weight gain (g)	905.60^b^	969.60^a^	928.40^b^	917.20^b^	7.20	0.002
Feed conversion ratio	1.33	1.29	1.30	1.30	0.02	0.897
Mortality (%)	6.67	1.67	1.67	5.00	1.75	0.713
Grower period (22 to 42 d)						
Feed intake (g)	2,468.60	2,506.20	2,561.40	2,558.00	36.87	0.807
Body weight gain (g)	1,315.80^ab^	1,293.40^b^	1,366.80^ab^	1,396.00^a^	14.47	0.032
Feed conversion ratio	1.87	1.94	1.87	1.83	0.03	0.625
Mortality (%)	9.09	0.00	0.00	1.81	1.87	0.275
Overall period (1 to 42 d)						
Feed intake (g)	3,787.80	3,756.60	3,807.80	3,824.20	64.18	0.987
Body weight gain (g)	2,221.20	2,263.00	2,295.20	2,313.20	16.32	0.208
Feed conversion ratio	1.60	1.61	1.59	1.57	0.02	0.830
Mortality (%)	15.75	1.67	1.67	6.81	3.21	0.384

SEM, standard error of the mean.

1) CON, basal diet only; MOS, basal diet supplemented with commercial oligosaccharides; CMM, basal diet supplemented with crude oligosaccharides extract from coconut milk meal; ST, basal diet supplemented with crude oligosaccharides extract from spent tea leaves.

a,b Means within a row with different letters differ significantly at p<0.05.

**Table 3:** Carcass and visceral organs weight in 42-d-old broiler chickens fed diets supplemented with crude oligosaccharides extract from agricultural by-products (n = 5)

Items	Dietary group^1)^				SEM	p-value

	CON	MOS	CMM	ST		
Carcass and visceral organs weight (g/100 g body weight)						
Carcass	88.13	87.21	86.74	88.03	0.36	0.494
Thigh+drumstick	1.20	1.17	1.15	1.15	0.01	0.700
Breast	1.33	1.18	1.28	1.34	0.03	0.279
Wing	0.45	0.43	0.44	0.43	0.01	0.883
Abdominal fat	1.12	1.08	1.13	1.19	0.07	0.972
Liver	1.49	1.63	1.59	1.52	0.04	0.678
Proventriculus	0.28	0.30	0.32	0.30	0.01	0.792
Gizzard	1.31	1.19	1.36	1.44	0.03	0.152
Heart	0.36	0.40	0.44	0.41	0.01	0.155

SEM, standard error of the mean.

1) CON, basal diet only; MOS, basal diet supplemented with commercial oligosaccharides; CMM, basal diet supplemented with crude oligosaccharides extract from coconut milk meal; ST, basal diet supplemented with crude oligosaccharides extract from spent tea leaves.

**Table 4:** Weight and length of duodenum, jejunum, ileum, and ceca in 42-d-old broiler chickens fed diets supplemented with crude oligosaccharides extract from agricultural by-products (n = 5)

Items	Dietary group^1)^				SEM	p-value

	CON	MOS	CMM	ST		
Intestinal weight (g/100 g BW)						
Duodenum	0.31	0.28	0.32	0.29	0.01	0.757
Jejunum	0.59	0.61	0.70	0.62	0.02	0.328
Ileum	0.52	0.50	0.48	0.50	0.01	0.872
Ceca	0.24	0.24	0.23	0.22	0.01	0.863
Intestinal length (cm/100 g BW)						
Duodenum	1.07	0.98	1.15	0.98	0.02	0.075
Jejunum	2.66	2.63	2.55	2.64	0.07	0.966
Ileum	2.39	2.55	2.37	2.61	0.10	0.832
Ceca	1.35	1.37	1.16	1.29	0.03	0.119

SEM, standard error of the mean; BW, body weight.

1) CON, basal diet only; MOS, basal diet supplemented with commercial oligosaccharides; CMM, basal diet supplemented with crude oligosaccharides extract from coconut milk meal; ST, basal diet supplemented with crude oligosaccharides extract from spent tea leaves.

**Table 5:** Villus height, villus area, crypt depth and villus height/crypt depth ratio of duodenum, jejunum, and ileum in 42-d-old broiler chickens fed diets supplemented with crude oligosaccharides extract from agricultural by-products (n = 5)

Items	Dietary group^1)^				SEM	p-value

	CON	MOS	CMM	ST		
Villus height (mm)						
Duodenum	1.38	1.44	1.41	1.59	0.04	0.345
Jejunum	1.03	1.06	1.08	1.18	0.04	0.701
Ileum	0.75	0.76	0.86	0.81	0.02	0.492
Villus area (mm^2^)						
Duodenum	0.18	0.18	0.18	0.23	0.01	0.356
Jejunum	0.13	0.12	0.11	0.13	0.01	0.758
Ileum	0.07	0.07	0.07	0.08	0.01	0.736
Crypt depth (mm)						
Duodenum	0.19	0.16	0.19	0.16	0.01	0.085
Jejunum	0.16	0.15	0.17	0.15	0.01	0.139
Ileum	0.14	0.15	0.16	0.13	0.01	0.628
Villus height/crypt depth ratio						
Duodenum	7.34	9.08	7.20	9.82	0.42	0.051
Jejunum	6.45	7.20	6.26	7.97	0.31	0.195
Ileum	5.46	5.38	5.44	5.93	0.24	0.867

SEM, standard error of the mean.

1) CON, basal diet only; MOS, basal diet supplemented with commercial oligosaccharides; CMM, basal diet supplemented with crude oligosaccharides extract from coconut milk meal; ST, basal diet supplemented with crude oligosaccharides extract from spent tea leaves.

**Table 6:** Nutrient digestibility in broiler chickens fed diets supplemented with crude oligosaccharides extract from agricultural by-products (n = 4)

Items	Dietary group^1)^				SEM	p-value

	CON	MOS	CMM	ST		
Nutrient digestibility (d 21)						
Dry matter (%)	68.54	76.20	67.67	71.66	1.22	0.052
Gross energy (%)	72.45	79.05	72.69	76.10	1.08	0.091
Nutrient digestibility (d 42)						
Dry matter (%)	55.28	64.85	70.32	64.19	2.15	0.089
Gross energy (%)	61.07^b^	70.37^ab^	75.09^a^	71.19^ab^	1.86	0.041

SEM, standard error of the mean.

1) CON, basal diet only; MOS, basal diet supplemented with commercial oligosaccharides; CMM, basal diet supplemented with crude oligosaccharides extract from coconut milk meal; ST, basal diet supplemented with crude oligosaccharides extract from spent tea leaves.

a,b Means within a row with different letters differ significantly at p<0.05.
